# Lightweight Transformer Model for Mobile Application Classification

**DOI:** 10.3390/s24020564

**Published:** 2024-01-16

**Authors:** Minju Gwak, Jeongwon Cha, Hosun Yoon, Donghyun Kang, Donghyeok An

**Affiliations:** 1 Department of Computer Engineering, Changwon National University, Changwon 51140, Republic of Korea; minju990222@gmail.com (M.G.); jcha@gs.cwnu.ac.kr (J.C.); 2 Network Research Department, Electronics and Telecommunications Research Institute, Daejeon 34129, Republic of Korea; yhs@etri.re.kr; 3 Department of Computer Engineering, College of IT Convergence, Gachon University, Seongnam-si 13120, Republic of Korea

**Keywords:** transformer model, application classification, wireless LAN, deep learning

## Abstract

Recently, realistic services like virtual reality and augmented reality have gained popularity. These realistic services require deterministic transmission with end-to-end low latency and high reliability for practical applications. However, for these real-time services to be deterministic, the network core should provide the requisite level of network. To deliver differentiated services to each real-time service, network service providers can classify applications based on traffic. However, due to the presence of personal information in headers, application classification based on encrypted application data is necessary. Initially, we collected application traffic from four well-known applications and preprocessed this data to extract encrypted application data and convert it into model input. We proposed a lightweight transformer model consisting of an encoder, a global average pooling layer, and a dense layer to categorize applications based on the encrypted payload in a packet. To enhance the performance of the proposed model, we determined hyperparameters using several performance evaluations. We evaluated performance with 1D-CNN and ET-BERT. The proposed transformer model demonstrated good performance in the performance evaluation, with a classification accuracy and F1 score of 96% and 95%, respectively. The time complexity of the proposed transformer model was higher than that of 1D-CNN but performed better in application classification. The proposed transformer model had lower time complexity and higher classification performance than ET-BERT.

## 1. Introduction

Since the COVID-19 epidemic, non-face-to-face activities have become more prevalent, leading to a surge in popularity for network-based real-time online services like video conferencing programs, augmented reality (AR), and virtual reality (VR). Furthermore, the demand for virtual environments where numerous real-life activities can be performed has also increased. In particular, the metaverse is gaining popularity because it enables economic and social activities that are not constrained by time or geography. By 2030, the market for virtual environments such as the metaverse, valued at USD 38.85 billion in 2021, is anticipated to grow at an average annual rate of 39.4%, reaching USD 772.24 billion [1]. To support hyper-realistic services such as the metaverse, real-time online services, and AR/VR applications, deterministic transmission with end-to-end ultra-low latency and high reliability is required.

To offer deterministic transmission, technological development across a variety of network stacks is necessary. At the data link layer, the wireless time-sensitive network (TSN) has been introduced to provide ultra-low latency and high-reliability transmission in WLAN and 5G [2]. Techniques such as orthogonal frequency division multiple access (OFDMA) are used to facilitate wireless TSN [3,4,5] in WLAN. The 3rd Generation Partnership Project (3GPP) has proposed 5G technologies for TSN to provide ultra-low latency and high reliability in mobile networks. For integration with TSN, the 5G system (5GS) defines translator functionality [6]. Periodic deterministic QoS and time synchronization provided by 5GS support scheduled traffic and per-stream filtering and policing (PSFP), as described by IEEE 802.1Q. For hyper-realistic service at the transport layer, several schemes have been proposed to provide partial reliability, application-aware data forwarding, and multi-path transmission. Reliability is ensured by retransmission offered by TCP or QUIC; however, this introduces additional transmission latency. Partial reliability performs selective retransmission depending on the data deadline, decreasing transmission delay by avoiding retransmission of pointless data [7,8]. For processing efficiency improvement, burst forwarding transmits data in units handled by applications rather than path MTU enabling simultaneous utilization of several processors and reducing contention overhead [9]. As a multi-path retransmission mechanism, XLINK suggests a priority-based re-injection technique to decrease latency [10]. If MPTCP, a representative multipath transmission protocol, encounters sporadic disconnection and reconnection, a quick re-injection approach is suggested for fast retransmission [11].

Although several schemes have been proposed to achieve low latency and high reliability, practical challenges exist in implementing the schemes to real services because each real-time application has different network performance requirements. Table 1 lists the network specifications for each realistic service application, including downlink and uplink bandwidth, latency, and transmission reliability [12]. Cloud gaming requires a delay of 10 to 30 ms, whereas VR and AR require a latency of 5 to 20 ms. Cloud gaming and VR require less uplink bandwidth than 2 Mbps, but AR needs an uplink bandwidth of 2–20 Mbps. Cloud gaming, VR, and AR demand more downlink than uplink bandwidth, but download bandwidth requirements for AR, cloud gaming, and VR are 2–60 Mbps, 8–30 Mbps, and 30–100 Mbps, respectively. All services require transmission reliability of 99.9% or greater. For real-time applications to operate successfully, different network requirements must be satisfied for each application. Using technologies like network function virtualization (NFV), the network core can deliver customizable performance for each application. Therefore, application classification is required to support network performance for each application.

Real-time application users prefer not to disclose application information they are using for application classification to network administrators due to privacy concerns. As as result, applications have been categorized based on network traffic. Traditionally, the information in the packet header such as port number has been used for this purpose, but this method potentially exposes personal information from the packet header [13]. If the packet header information is encrypted to protect personal information, categorizing the application becomes impossible because the encrypted information cannot be used. Regardless of whether the packet header information is encrypted or not, artificial intelligence is used to categorize applications.

To categorize applications using deep learning, models such as ET-BERT and 1D-CNN have been proposed [14,15]. Both models can categorize applications even when packet header information is encrypted. However, the risk of personal information disclosure still exists because encrypted header information is used. Therefore, it is crucial to achieve application classification using application data without relying on a header. CNN can be used for traffic classification, but it should be supplemented with other methods, such as long short-term memory (LSTM) or ant-lion meta-heuristic algorithm (ALO) to obtain good classification performance. ET-BERT, which uses multilayer bidirectional transformer blocks, requires substantial computational resources for application classification. Given that applications have a simpler structure than natural languages and require faster classification performance, the complexity of ET-BERT increases, but the classification performance remains constant.

In this study, for application categorization, we aimed to propose a lightweight transformer model that meets the following requirements. The requirements are as follows. (1) We use encrypted payload omitting encrypted or unencrypted header. (2) We achieve low computational overhead. First, in the wireless LAN, we gathered wireless packets from four different applications: Instagram, Tving, Netflix, and YouTube. The data were preprocessed to omit header data before being used as model input. To classify applications, we proposed a lightweight transformer model composed of an encoder, a pooling layer, and a dense layer. Finally, we evaluated the performance of our proposed model and compared it with other deep learning models, such as 1D-CNN and ET-BERT [14,15]. The proposed model achieved a classification accuracy of over 96% in the evaluation results, outperforming the other models.

The remainder of this paper is organized as follows: In Section 2, we present background and related works. In Section 3, we discuss the dataset collected from four applications in WLAN and propose a transformer model for mobile application classification. In Section 4, we evaluate the performance of the proposed application classification model. Finally, Section 5 concludes the paper.

## 2. Background and Related Works

Application classification using machine learning has long been researched. The most frequently utilized features were flow features, such as flow duration and flow bytes per second, packet features, such as packet size and inter-packet duration, or combinations of flow and packet features [16,17,18,19,20]. Recently, deep learning methodologies have been used for application classification [14,15,21,22,23]. Convolution neural networks (CNNs) and transformers among deep learning models have good accuracy in classification applications.

A convolutional neural network (CNN) is a neural network that simulates the structure of the human optic nerve. A kernel (filter) can be used to generate feature maps, and kernel learning can improve feature map extraction. Consequently, a 2D-CNN performs well for object identification or image classification, and a 1D-CNN performs well for text classification in natural language processing. In [14], 1D-CNN is also utilized in application categorization due to the structure of network traffic, which is composed of bytes, packets, and flows, comparable to natural language patterns such as characters, words, and sentences. The 1D-CNN model consists of three phases: preprocessing, training, and test phases. The preprocessing phase proceeds with traffic split, traffic cleaning, image generation, and IDX conversion. Traffic split divides the traffic based on session and flow, and the first 784 bytes of the traffic are extracted through traffic cleaning. The retrieved traffic is then transformed into an idx3 format for image generation. The idx3 data produced during the training phase are used for mini-batch stochastic gradient descent (SGD) training. The performance of the trained model is assessed during the test phase. In [24], end-to-end representation learning is used to classify network traffic. First, LSTM is utilized to examine the temporal dependency of traffic. Then, to classify traffic, local patterns are extracted using CNN. In [25], network traffic types such as chat, ftp, and mail are identified using a combination of CNN, ALO, and a self-organized map (SOM). CNN is used to extract network traffic features, and ALO chooses the features required to determine the traffic type. SOM is used to determine traffic type based on the selected features. To improve classification performance, CNN studies involve image conversion from network traffic and integration with other techniques. The proposed model, however, requires payload extraction and does not require the use of other techniques.

The transformer model [26], which is based on the attention mechanism and does not require recurrent neural networks (RNN) or CNN, presents improved performance in translation. The encoder–decoder structure is used in the transformer model. Positional encoding is used to add location information as the attention mechanism does not store it for each input token. The encoder and decoder of the transformer model utilizes three multihead attentions with scaled dot-product attention serving as their core component. Scaled dot-product attention indicates the association between a given query and key. For this purpose, the inner product of the query and key vectors is applied to the softmax function. To produce an attention value vector that represents the association for each word, the result of the softmax function is multiplied by the value vector. The multihead attention of the encoder and decoder conducts H-scaled dot-product attention, sums each vector, and outputs a value multiplied by a weight matrix. To prevent references to padding values or future data, the masked multihead attention of the decoder performs a mask on the scaled dot-product attention.

Bidirectional encoder representations from the transformer (BERT), which uses a transformer encoder, is a pre-trained model that can comprehend language representations using a sizable text corpus [27]. By fine-tuning the pre-trained models, tasks such as word prediction, problem generation, and sentiment analysis can be performed. A pretrained model called ET-BERT was developed by applying a BERT-like technique to packets [15]. ET-BERT employs two unsupervised learning techniques for learning packet representations: one that involves masking 15% tokens of the input sequence and predicting the mask tokens, and another that involves determining whether the same application packet is present. ET-BERT encodes a packet as a hexadecimal sequence composed of two consecutive bytes to tokenize the byte data in the packet. The model size of the ET-BERT base is the same as BERT, and the network structure of ET-BERT is composed of multilayer bidirectional transformer blocks. However, due to the enormous size of the ET-BERT model, the computational cost is considerable; however, the proposed model has a lightweight structure, and therefore, the computational overhead is low.

## 3. Proposed Scheme

In this section, we describe the dataset gathered for application classification, data preprocessing, and the structure of the suggested transformer model.

### 3.1. Dataset

**Figure 1:**
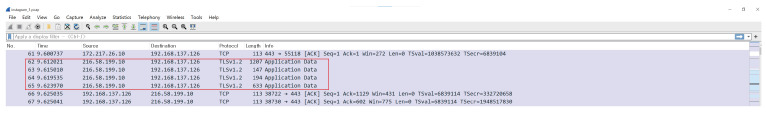
Screenshot for dataset collection.

We gathered mobile traffic from a total of four applications: YouTube, Netflix, Instagram, and Tving. Tving is the leading over-the-top (OTT) streaming service in Korea. Instagram, Netflix, and YouTube are well-established international services. From December 2020 to March 2021, we constructed and ran a wireless LAN environment as well as gathered wireless LAN traffic using Wireshark [28]. To produce the dataset, the application traffic of smart devices with Android or iOS was categorized using MAC address-based filtering, and only application traffic was extracted based on IP address and port number. Figure 1 displays a screenshot of the data collection process. We ensured that no personal information was used because the collected application data were encrypted using TLS, as indicated in the figure, and headers are excluded from the collected traffic. Application data packets for YouTube, Netflix, Instagram, and Tving were extracted from the collected data. In total, 430,920 packets totalling 558 MB were gathered for four applications, as listed in Table 2. The number and total size of packets for each application were adjusted equally to avoid bias when learning the application classification model based on the transformer.

### 3.2. Data Preprocessing

**Figure 2:**
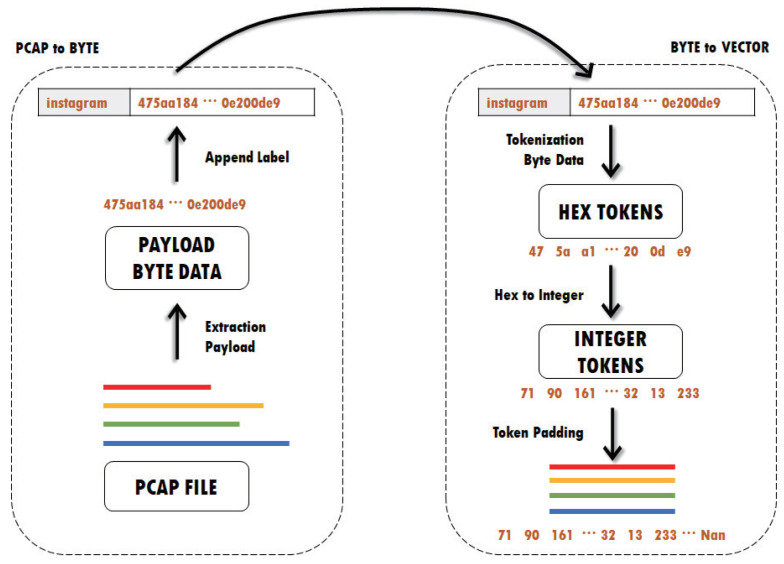
Data preprocessing.

**Figure 3:**
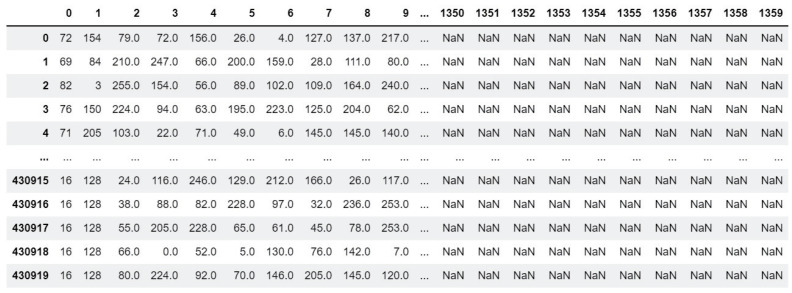
Example of token padding.

We preprocessed the dataset because the gathered traffic includes a payload and several headers. As the payload is encrypted using TLS, personal information is protected. However, headers can expose personal information. Therefore, we conducted preprocessing to utilize the encrypted application payload without headers. Figure 2 illustrates the two phases of dataset preprocessing: extracting payload bytes from collected packets and transforming them into input vectors. First, we used Scapy to decode the packets stored in the pcap file, as the traffic was captured using Wireshark [29]. From the collected packets, we removed headers such as MAC, IP, TCP, and etc., and extracted the payload. Each payload then had a label applied. The second step is to tokenize the payload into 2 bytes and then convert each token to an integer. Because the payload has a variable length, the payload size must be modified to be the same for the transformer model. For all payload lengths, the mode is determined during the transformation of tokens into input vectors, and the mode value was 1360 in this study. If the payload is shorter than the mode, it is padded to match the length of the mode, as depicted in Figure 3. NaN represents the padded part in the figure that corresponds to the mode. If the payload length exceeds the mode, the surplus portion of the payload is excluded.

### 3.3. Transformer Model

**Figure 4:**
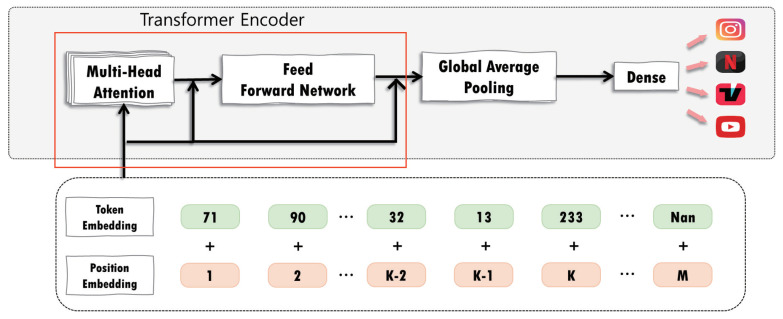
Architecture of the transformer model.

For application classification, we employed a transformer model. The architecture and structure of the proposed transformer model are depicted in Figure 4 and Table 3, respectively. Input embedding is performed on preprocessed data to produce inputs for the proposed model. Input embedding employs input and output vectors with a dimension of 1360 × 1. Position embedding is used to add positional information to tokens. Token embedding is then performed to produce an input vector with 512 fixed-size dimensions. For position embedding and token embedding, the token and position embedding layer was employed, and an output vector with a dimension of 1360 × 512 was produced. Because packets have a simpler structure than natural language, the proposed model uses one transformer encoder to reduce complexity. The encoder, which is composed of a multihead attention and feed-forward network, receives the vector obtained during the embedding process as an input vector. The transformer encoder consists of two components: multihead attention and a feed-forward network. Multihead attention is composed of four layers, each of which performs an attention process. The attention vector is obtained by applying the attention score to the softmax function and multiplying the outcome by the value vector. The attention vectors calculated for each layer are concatenated to provide the multihead attention result, which is a vector with a dimension of 1360 × 512. The feed-forward network includes two fully connected dense layers, each containing 512 nodes. The GeLU activation function is employed after the first dense layer. To prevent gradient vanishing, residual connection and layer normalization are performed after multihead attention and feed-forward network. The transformer encoder produces a vector of the dimensions 1360 × 512. While ET-BERT employs 12 transformer encoders with 12 heads [15], we employ one transformer encoder with four heads for the lightweight transformer model. Global average pooling was used to reduce the dimensionality of the output value of the feed-forward network, resulting in an output vector with dimension of 512 × 1. The output value of the pooling layer is utilized as the input for a dense layer that classifies applications. The dense layer uses the softmax function as an activation function to describe probability for each of the four application types.

## 4. Evaluation

In this section, we assessed the performance of the proposed transformer model. First, we present the metrics used to assess the performance of the proposed model. Then we determine hyperparameters including the learning rate, epoch, and the number of feed-forward networks to enhance the performance of the proposed model. Finally, we compare the performance of the proposed model to those of ID-CNN and ET-BERT.

We evaluated the performance of the model using four metrics: accuracy, precision, recall, and F1 score. Accuracy is the percentage of accurate forecasts among all predictions, as demonstrated by Equation (1).

(1) $$Accuracy=\frac{TP+TN}{TP+TN+FP+FN}$$

where TP (true positive) and TN (true negative) represent actual positive and negative values, respectively, which are accurately predicted to be positive and negative. FP (false positive) represents an actual negative value that is mistakenly forecasted as positive, and FN (false negative) represents an actual positive value that is mistakenly anticipated as negative. For instance, TP predicts a YouTube packet as emerging from YouTube, whereas FP incorrectly predicts a YouTube packet as not originating from YouTube. In addition, TN predicts a non-YouTube packet as not originating from YouTube, and FN incorrectly predicts a non-YouTube packet as originating from YouTube. According to Equation (2), precision is defined as the ratio of actual positives to predicted positives.

(2) $$Precision=\frac{TP}{TP+FP}$$

In Equation (3), the proportion of expected positives to actual positives is referred to as recall.

(3) $$Recall=\frac{TP}{TP+FN}$$

The F1 score, which is the harmonic average of precision and recall, is used to consider both precision and recall simultaneously. The equation for the F1 score is as follows:

(4) $$F1score=\frac{2\times Precision\times Recall}{Precision+Recall}$$

**Figure 5:**
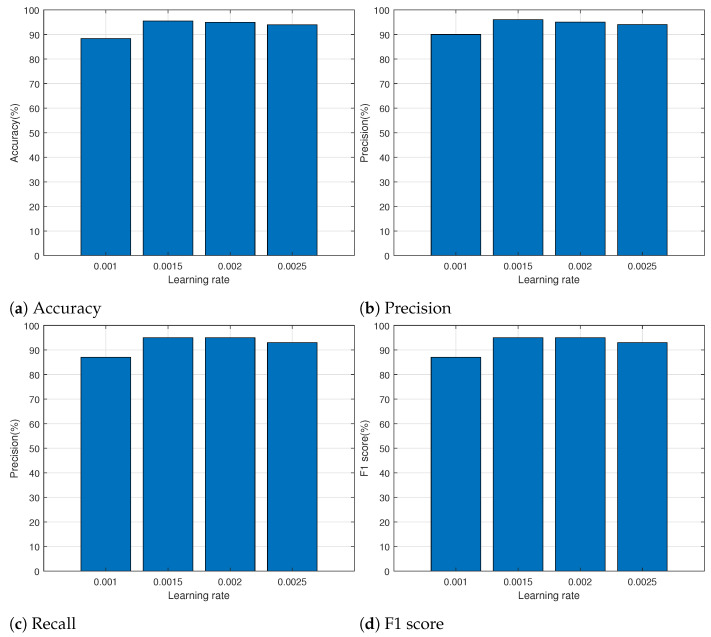
Performance measurement for each learning rate.

Here, the proposed model was evaluated on a server with a Xeon CPU, 128GB RAM, and two GTX 1080 Ti GPUs. The operating system was Ubuntu 20.04 LTS, and the framework was Tensorflow 2.5.0 with CUDA version 11.04. We determine the hyperparameters such as learning rate, epoch, and etc. to enhance the performance of the proposed model. Table 4 specifies the hyperparameters for the proposed transformer model. In the table, the number of feed-forward networks and the number of heads indicate the number of nodes in the feed-forward network sub-layer and the number of heads in multihead attention, respectively. The embedding dimension denotes the input size for multihead attention. We determined the learning rate by performing evaluations for each learning rate to enhance the performance of the proposed model. Figure 5 shows the results of measuring accuracy, precision, recall, and F1 score while increasing the learning rate by 0.0005 from 0.001 to 0.0025. We used the sklearn.metrics module to obtain the evaluation results for each performance metric [30]. The results showed that the learning rate of 0.001 performed the worst across all metrics. The performance of all measuring metrics was at its peak when the learning rate was 0.0015, and it declined as the learning rate increased. Therefore, the learning rate for this study was set at 0.0015.

**Figure 6:**
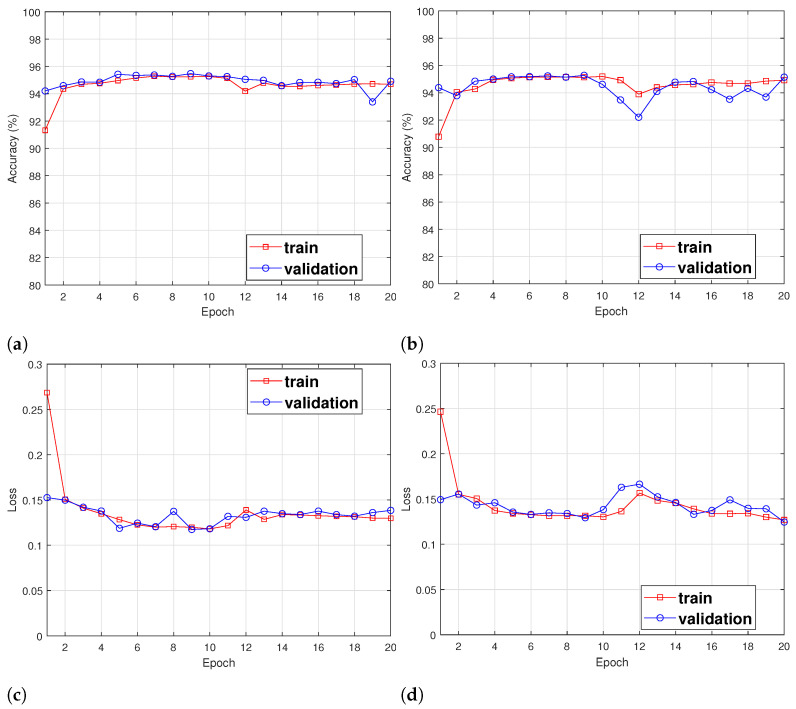
Performance measurement for each epoch with different nodes of feed-forward network. (**a**) Accuracy with 512 nodes of feed-forward network; (**b**) accuracy with 1024 nodes of feed-forward network; (**c**) loss with 512 nodes of feed-forward network; (**d**) loss with 1024 nodes of feed-forward network.

**Figure 7:**
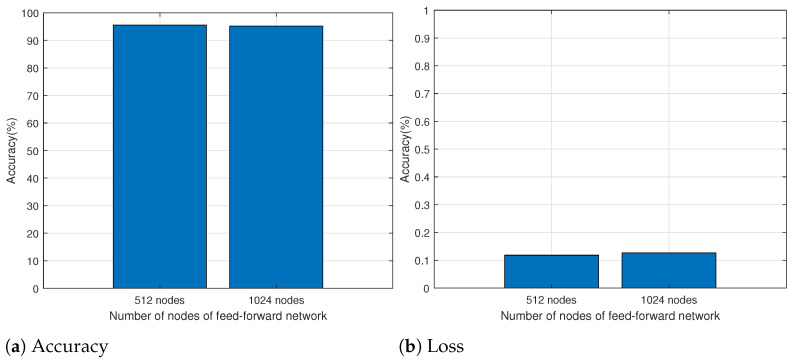
Performance measurement for different nodes of feed-forward network.

Then, we measured the accuracy and loss of the training and validation datasets for each epoch. The results are shown in Figure 6. Figure 6a represents the measurement results of the accuracy for each epoch. Despite sporadic fluctuations after the fifth epoch, the accuracy for the training and validation datasets is relatively stable. The loss for each period is shown in Figure 6c. In contrast to accuracy, the training and validation loss experiences fluctuation, and the loss for both the training and validation datasets stabilizes from the 15th epoch onwards. Therefore, the epoch for this study was set at 20. We evaluated performance with various numbers of nodes to determine the number of nodes in the feed-forward network. Figure 6b,d show the accuracy and loss for each epoch when the number of nodes in the feed-forward network is 1024. As shown in the figures, the accuracy and loss at the 20th epoch are similar to those observed when the feed-forward network consists of 512 nodes. However, the accuracy of the validation dataset remains variable, and the loss function is unstable for both the training and validation datasets. We compared the performance of feed-forward networks with 512 and 1024 nodes. Figure 7 represents the results. When using 512 nodes and 1024 nodes, the accuracy is approximately 95.5% and 95.1%, respectively. The loss for 512 and 1024 nodes is approximately 0.118 and 0.126, respectively. Even if the number of nodes in the feed-forward network increases, there is no gain in accuracy and loss, and the complexity of the proposed model increases as the number of nodes increases. Therefore, we set the number of nodes in the feed-forward network at 512.

**Figure 8:**
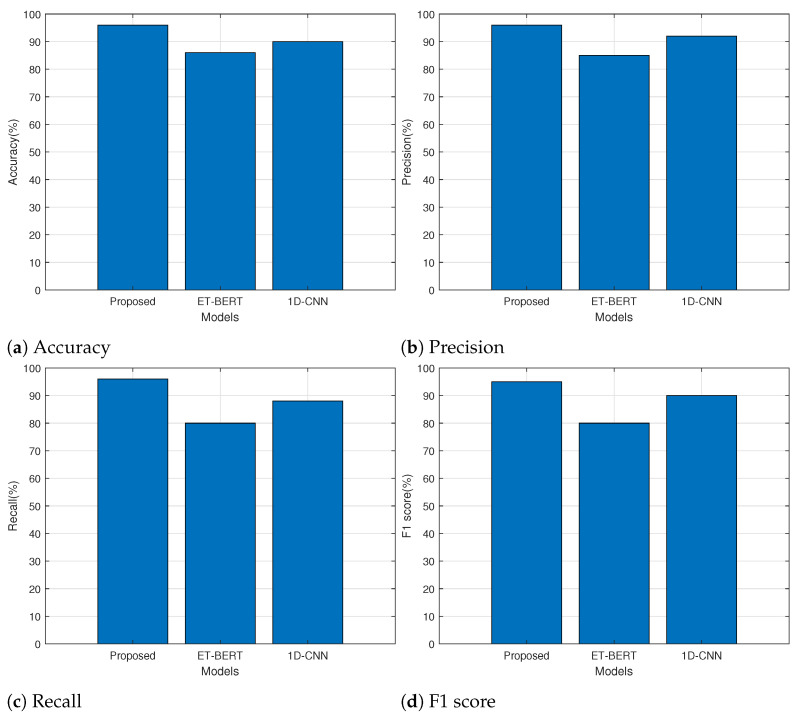
Performance comparison for each model.

We compared the performance of the proposed transformer model with those of the 1D-CNN and ET-BERT models. We implemented the 1D-CNN and ET-BERT models with parameters specified in [14,15]. We measured the performance of 1D-CNN and ET-BERT using the sklearn.metrics module and the evaluation function of ET-BERT, respectively, [30,31]. Each model’s accuracy, precision, recall, and F1 score are shown in Figure 8. The accuracy of successfully classifying the application among all predictions is shown in Figure 8a. The accuracy of the proposed model is 96% compared to 86% and 90% for ET-BERT and 1D-CNN, respectively. Precision indicates the percentage of correctly predicted results among all the results predicted for a specific application. The precision results for each model are shown in Figure 8b. With a precision of 96%, the proposed model outperformed the other two models. Recall indicates the percentage of applications that can be reliably predicted based on the traffic of a particular application. The results shown in Figure 8c demonstrate that the recall performance of the ET-BERT and 1D-CNN models was less than 90%, whereas the recall performance of the proposed model was 96%. The proposed model, with an F1 score of 95%, demonstrated the highest classification performance, as shown in Figure 8d, whereas ET-BERT and 1D-CNN had F1 values of 80% and 90%, respectively. For application classification, ET-BERT and 1D-CNN require both the payload and the header. However, because the proposed model uses the application payload, it outperforms the other two models in performance evaluation.

In addition to the four metrics of accuracy, precision, recall, and F1 score, practicability is an important factor in evaluating model performance. Assuming the input size of n, the time complexity of the transformer and CNN is $O(n^{2})$ and O(n), respectively, [26,32]. Because the proposed model and ET-BERT theoretically have the same time complexity, we measured the execution time for training and evaluation to compare the time complexity of the proposed transformer model with other models. Table 5 shows the measurement results. Because the transformer has a more complex model structure than CNN, ET-BERT and the proposed model requires longer training time than 1D-CNN. However, because image conversion is required, 1D-CNN requires more time than the measured training time. Since the proposed model has a lighter structure than ET-BERT, one epoch of the proposed model takes around 2695 s, whereas ET-BERT takes about 4674 s. The evaluation time of the proposed model is approximately 451 s, which is longer than 1D-CNN but shorter than ET-BERT. However, the proposed model outperformed the others in classification performance. If the dataset is increased for higher classification accuracy, not only the classification accuracy but also the execution time of training and evaluation will increase, and only time complexity will increase after obtaining the maximum accuracy. As a result, the appropriate dataset size will vary based on the number of applications to be classified and the quality of the dataset.

**Figure 9:**
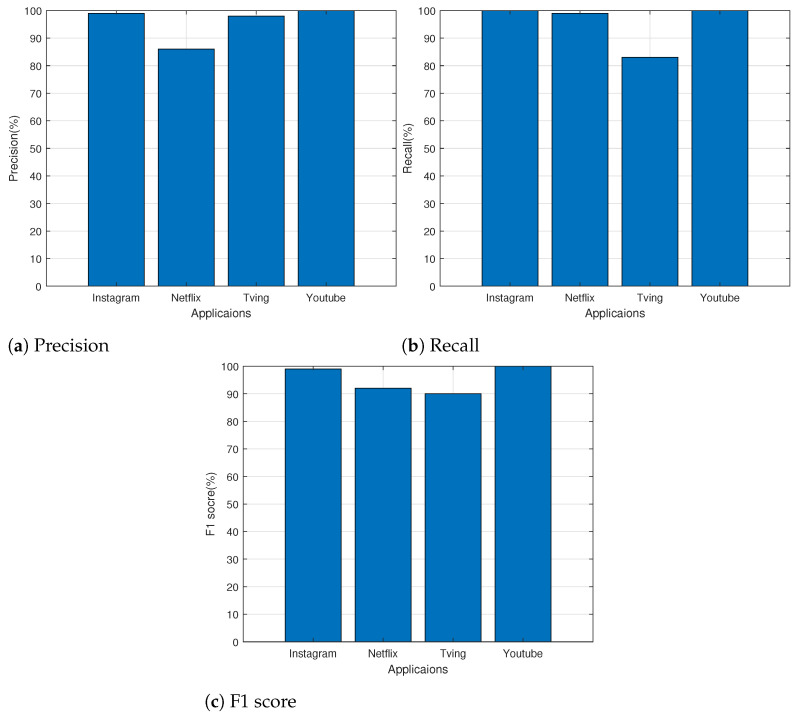
Classification performance for each application.

We analyzed the classification performance of the proposed model in terms of application. Figure 9 shows the measured precision, recall, and F1 score for each application. According to the results, Instagram and YouTube performed between 99% and 100% across all metrics. This indicates that the proposed transformer model appropriately classifies YouTube and Instagram applications. With an F1 score of 92% and 90% for Netflix and Tving, respectively, the proposed model demonstrated good classification ability. However, compared to Instagram and YouTube, the classification performance of the suggested model is relatively poor for the Netflix and Tving applications. When classifying applications based on traffic, the traffic of other applications may be incorrectly determined to be their traffic because Netflix and Tving, which are OTT service applications, belong to the same service category. Compared to Tving, Netflix has a high recall value and a low precision value. This result indicates that traffic from Netflix is correctly categorized as Netflix, but some traffic from Tving, a non-Netflix application, is categorized as Netflix. In contrast, because some of the traffic on Tving is categorized as Netflix, Tving has a low recall value and high precision.

## 5. Conclusions

We proposed a lightweight transformer model for classifying applications based on traffic. To construct a dataset for application classification, we collected encrypted traffic from four well-established applications. During data preprocessing, we removed headers and extracted the encrypted application data from the collected traffic, ensuring personal information protection, and converted it into model input. We proposed a transformer model with a transformer encoder, a pooling layer, and a dense layer to achieve a lightweight model structure. Various evaluations were performed to determine the hyperparameters. In terms of application classification, the proposed transformer model outperformed both 1D-CNN and ET-BERT.

## Figures and Tables

**Table 1:** Network specifications for each realistic service application [12].

	Downlink (Mbps)	Uplink (Mbps)	Latency (ms)	Reliability (%)
Cloud gaming	8–30	0.3	10–30	≥99
VR	30–100	<2	5–20	≥99
AR	2–60	2–20	5–50	≥99

**Table 2:** Number and total size of packets gathered by applications.

Application	The Number of Packets	Size (MB)
Instagram	130,972	148
Netflix	100,000	151
Tving	100,000	135
Youtube	99,948	124
Total	430,920	558

**Table 3:** Structure of the transformer model.

Model Layer		Input Vector	Output Vector
Input layer		1360 × 1	1360 × 1
Token and position embedding		1360 × 1	1360 × 512
Transformer block	Multihead attention (head = 4)	1360 × 512	1360 × 512
	Feed forward network	1360 × 512	1360 × 512
Gloval average pooling		1360 × 512	512 × 1
Dense		512 × 1	4 × 1

**Table 4:** Hyperparameters for the proposed model.

Hyperparameters	Values
batch size	64
epoch	20
number of feed-forward networks	512
number of head	4
embedding dimension	512
dropout	0.3
learning rate	0.0015

**Table 5:** Execution time measurement.

	Proposed	ET-BERT	1D-CNN
training	2695.2	4674.3	70.0
evaluation	451.2	794.7	25.5

## Data Availability

Data are contained within the article.

## Abbreviations

The following abbreviations are used in this manuscript:

AR	Augmented reality
VR	Virtual reality
TSN	Time sensitive network
OFDMA	Orthogonal frequency division multiple access
3GPP	3rd Generation Partnership Project
5GS	5G system
PSFP	Per-stream filtering and policing
ABR	Adaptive bit rate
NFV	Network function virtualization
CNN	Convolution veural network
SGD	Stochastic gradient descent
RNN	Recurrent neural network
BERT	Bidirectional encoder representations from transformer
OTT	Over the top
LSTM	Long short term memory
ALO	Ant-lion meta-heuristic algorithm
SOM	Self-organized map
